# Altered activation patterns of sensorimotor cortex during level walking and stair climbing in patients with knee osteoarthritis: a cross-sectional study

**DOI:** 10.3389/fnagi.2026.1847015

**Published:** 2026-08-19

**Authors:** Liming Jiang, Kun Yang, Tianzhong Zi, Yanuo Li, Jiayi Xia, Sizhong Wang

**Affiliations:** 1 Department of Rehabilitation, Seventh People’s Hospital of Shanghai University of Traditional Chinese Medicine, Shanghai, China; 2 School of Medicine and Health Sciences, Wuxi Taihu University, Wuxi, China; 3 Clinical Research Center, The Second Rehabilitation Hospital of Shanghai, Shanghai, China; 4 School of Exercise and Health, Shanghai University of Sport, Shanghai, China

**Keywords:** functional near-infrared spectroscopy, knee osteoarthritis, level walking, sensorimotor cortex, stair climbing, surface electromyography

## Abstract

**Background:**

Knee osteoarthritis (KOA) is characterized not only by peripheral joint pathology but also by mechanisms related to central sensitization. Previous studies have reported reduced activation of sensorimotor cortex during isolated joint movements in patients with KOA. However, cortical activation during walking remains unclear. This study aimed to investigate sensorimotor cortex activation during different walking tasks in patients with KOA to identify potential targets for central interventions.

**Methods:**

Nineteen patients with KOA and 18 demographically matched healthy controls (HCs) were recruited. Functional near-infrared spectroscopy (fNIRS) was used to monitor hemodynamic activity in bilateral primary motor cortex (M1), primary sensory cortex (S1), and somatosensory association cortex (SAC). Paired *t*-tests and mixed analysis of variance were performed to examine interhemispheric and between-group differences. Clinical assessments included pain intensity assessed by VAS and functional status assessed by WOMAC. Pearson correlation analyses were performed to examine associations between cortical activation and clinical scores.

**Results:**

HCs showed symmetrical bilateral cortical activation across tasks, whereas patients with KOA exhibited reduced cortical activation in the left hemisphere compared with the right. During stair tasks, activation in the left M1, S1, and SAC in patients with KOA was significantly lower than that in the right (*P* < 0.05). During level walking, only left S1 activation was significantly lower than the right (*P* < 0.05). Compared with HCs, patients with KOA also exhibited reduced activation in the left hemisphere, with significant lower activation in the left M1, S1, and SAC during stair tasks (*P* < 0.05), and reduced activation in the left S1 during level walking (*P* < 0.05). No significant between-group differences were observed in the right hemisphere. Additionally, activation in the left M1 and S1 was negatively correlated with VAS scores, and activation in the left M1 was negatively correlated with WOMAC function scores.

**Conclusion:**

Patients with KOA exhibit reduced contralateral sensorimotor cortex activation during walking tasks, with greater reductions as task difficulty increases. Lower activation in contralateral M1 and S1 is associated with higher pain and poorer functional status, highlighting the role of central mechanisms in KOA and potential targets for neuromodulatory interventions.

## 1 Introduction

Knee osteoarthritis (KOA) is a common chronic joint disorder marked by progressive cartilage degeneration, subchondral bone remodeling, synovial inflammation, and osteophyte formation (Kraus et al., 2015; Jeffrey et al., 2021; Hu et al., 2024). Patients with KOA often experience chronic pain, joint stiffness, functional limitations during weight-bearing activities, and progressive joint deformity, all of which substantially impair quality of life (Hunter and Bierma-Zeinstra, 2015; Liu et al., 2024). With the rapid aging of the global population, KOA has become a leading cause of disability worldwide and imposes an increasing healthcare and socioeconomic burden (Daniel et al., 2013; Palazzo et al., 2016; David et al., 2020; Yueming et al., 2025).

Current management strategies for KOA primarily involves pharmacotherapy, exercise, manual therapy, and surgical interventions (Philip, 2013; Alberto et al., 2021; Ling et al., 2023; Tianxiao et al., 2023; Mitchell et al., 2025). Although these approaches can relieve symptoms in some patients, they have limitations, including medication-related adverse effects, limited long-term effectiveness, and the invasiveness and cost associated with surgical interventions (Bill and Penny, 2013; Andrea et al., 2025). Moreover, these conventional strategies predominantly target peripheral joint pathology, such as inflammation and structural degeneration, while insufficiently attention is given to central nervous system (CNS) mechanisms involved in pain perception and motor control (Marwan et al., 2014; Cody et al., 2022). Growing evidence highlights that consider both peripheral pathology and central mechanisms contributing to the development of and persistence of KOA symptoms (Marylie et al., 2023; Kanlayanee et al., 2025; Wei-Ju et al., 2025; Yifan et al., 2025; Zahra Bagherpoor and Razieh, 2025).

A notable clinical feature of KOA is the discordance between radiographic severity and reported symptoms (Erlangga et al., 2010; Phillips and Clauw, 2013; Henri et al., 2025). Up to 50% of individuals with moderate-to-severe structural damage remaining asymptomatic, while approximately 10% reporting significant pain present with normal radiographs (Hannan et al., 2000). This mismatch suggests that pain in KOA cannot be fully explained by peripheral pathology alone, highlighting the role of central sensitization and altered CNS pain processing (Philip et al., 2011). Dysregulation of central pain modulatory pathways may therefore significantly shape the clinical pain experience, independent of or in addition to, structural joint damage (Marwan et al., 2014). Consequently, investigating central mechanisms is thus essential for advancing our understanding and treatment of KOA.

With advances in neuroimaging, increasing attention has been directed toward CNS alterations in patients with KOA and their relationship with pain and dysfunction (Marcel et al., 2022; Shirui et al., 2024; Yang et al., 2024). A study using magnetic resonance imaging (MRI) demonstrated that patients with KOA exhibited reduced gray matter volume (GMV) in critical areas such as the bilateral insula and hippocampus, regions associated with pain perception, emotional regulation, and memory processing (Kang et al., 2022). This reduction in GMV could be linked to the chronic pain experienced by these patients, as the severity of pain was found to correlate with functional alterations in the left fusiform gyrus and the extent of GMV loss in the left insula (Kang et al., 2022). Additionally, altered white matter integrity observed in patients with KOA, with diffusion tensor imaging revealing significant differences in fractional anisotropy and mean diffusivity across various white matter tracts (Cheng et al., 2022). The aforementioned findings suggest that KOA is associated with both gray matter and white matter abnormalities, which may contribute to the persistence of pain and functional impairment in these patients (Joaquín et al., 2023; Fuad et al., 2025).

Functional studies provide complementary insights. Altered brain activation patterns have been observed in patients with KOA, particularly during movement tasks that impose mechanical load and demand sensorimotor integration (Bishnoi et al., 2024; Yang et al., 2024). Meanwhile, previous study has observed altered contralateral cortical activation patterns in patients with KOA during affected knee joint movements (Yang et al., 2024). For example, our previous study using fNIRS revealed reduced activation in key sensorimotor regions on the contralateral side of the affected knee joint during isokinetic knee movements in patients with KOA, suggesting disrupted neural mechanisms for motor control and sensory processing (Yang et al., 2024). These abnormalities reflect the complex interplay between chronic pain, motor dysfunction, and central neuroplastic changes in KOA.

However, the aforementioned study was conducted under controlled laboratory conditions, which may not adequately reflect daily functional activities. In real-world contexts, such as level walking and stair climbing, patients often report greater pain and discomfort due to increased joint loading and biomechanical demands, which may further influence activity of CNS (Chen et al., 2021; Yosuke et al., 2023; Jing et al., 2025). Investigating cortical activation during these ecologically relevant tasks may therefore provide more clinically meaningful insights into CNS involvement in KOA.

Therefore, we aimed to investigate sensorimotor cortex activation in patients with KOA during functional walking tasks using functional near-infrared spectroscopy (fNIRS), including level walking, stair ascent and stair descent. Additionally, we aimed to examine the associations between cortical activation, pain intensity, and functional outcomes. Understanding these relationships may help identify potential neural targets for centrally oriented interventions in KOA.

### 1.1 Study design

This cross-sectional study enrolled participants with KOA along with demographically matched HCs, following the procedures outlined in a previously published protocol (Deng et al., 2022). This study was conducted in accordance with the Strengthening the Reporting of Observational Studies in Epidemiology guidelines to ensure transparent reporting (von Elm et al., 2008). Prior to participation, all participants were fully informed of the research aims and provided their written consent. Ethical approval was obtained from the Ethics Committee of Shanghai Seventh People’s Hospital (2024-7th-HIRB-098), and the study protocol was registered with the Chinese Clinical Trial Registry (ChiCTR2400092793).

### 1.2 Sample size calculation

We calculated sample size using an analysis of variance (ANOVA) design, comprising 2-group (between-group factor) × 3 measures (within-group factor: level walking, stair ascent, and stair descent), with the primary focus on the group × task interaction for brain activation between the two groups. Based on a prior study (Yang et al., 2024), our estimate were derived using an effect size of 0.22, a significance level of 0.05, power of 0.80. Therefore, a sample size of at least 36 participants was required to provide sufficient statistical power for this study. We calculated the sample size using G*Power 3.1.9.7 (University of Düsseldorf, Germany).

### 1.3 Participants

We recruited 19 patients with KOA via posters at the hospital’s outpatient clinic and local health centers, while 18 HCs via posters from nearby communities from June to August 2025. Inclusion criteria for the KOA group were: (1) aged 50–75 years; (2) meet the KOA diagnostic criteria based on the American College of Rheumatology’s clinical criteria; (3) the affected knee joint was only on the right side, and all patients had right leg dominance; (4) the kellgren-lawrence (KL) radiographic grade was II or III; (5) the pain score of knee joint ≥ 3 points (out of 10 points) for at least 3 months. Exclusion criteria were: (1) suffering from other arthritis such as rheumatoid arthritis; (2) history of severe knee injury; (3) history of neurological or psychiatric disorders; (4) drug/alcohol abuse; (5) cognitive impairment or inability to comply with study procedures. Inclusion criteria for the HC group were: (1) no history of joint or brain diseases; and (2) right leg dominant.

### 1.4 Experimental procedure

All tests were conducted in a quiet, controlled room to minimize external interference. Prior to the experiment, an experienced physical therapist collected demographic data from all participants and administered the Visual Analog Scale (VAS) for knee pain and the Western Ontario and McMaster Universities Osteoarthritis Index (WOMAC) for functional assessment. Following these, another professional tester explained the test procedures, movement tasks, and safety precautions to all participants. Adequate practice was allowed for all participants to become acquainted with the experimental procedures, thereby facilitating proficient execution during the actual session. Subsequently, the fNIRS optodes and surface electromyography (sEMG) electrodes were attached to each participant. Participants were tested in the order of their scheduled appointments.

**FIGURE 1:**
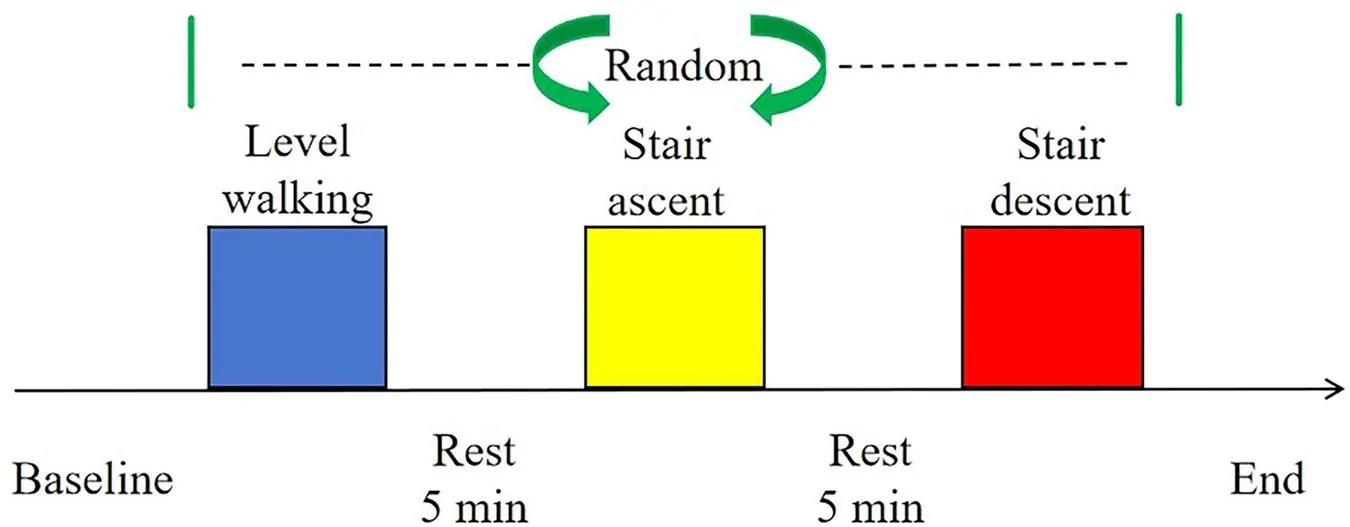
Flowchart for each testing session: three colors randomly indicate motor tasks (level walking, stair ascent, and stair descent).

The experimental protocol required participants to perform level walking, stair ascent, and stair descent tasks in a randomized sequence, with each task repeated three times. Before each test, a 30 s standing rest period was collected as baseline period. For level walking, participants walked for 30 s at a self-selected comfortable speed. For stair tasks, participants performed stair ascent and descent on a standardized staircase with 10 steps, each 12 cm in height, in accordance with the Chinese national standard, without using handrails. To control the speed-related confounding effects, HCs performed the same task at the patients’ average speed. After each test, a 30 s standing rest period was collected as a recovery period. A rest period of at least 5 min was allowed between tasks to ensure that the fNIRS signals returned to baseline levels. fNIRS data recording was synchronized with task execution through triggers built into the device. At the beginning of the baseline period, the beginning of the task, the end of the task, and the end of the recovery period for each task, researchers manually send event markers to the fNIRS data stream. Real-time sEMG signals were recorded from the participants’ lower limb muscles during the task performance. A simple flow of the experiment protocol is shown in Figure 1.

### 1.5 fNIRS data acquisition and preprocessing

**FIGURE 2:**
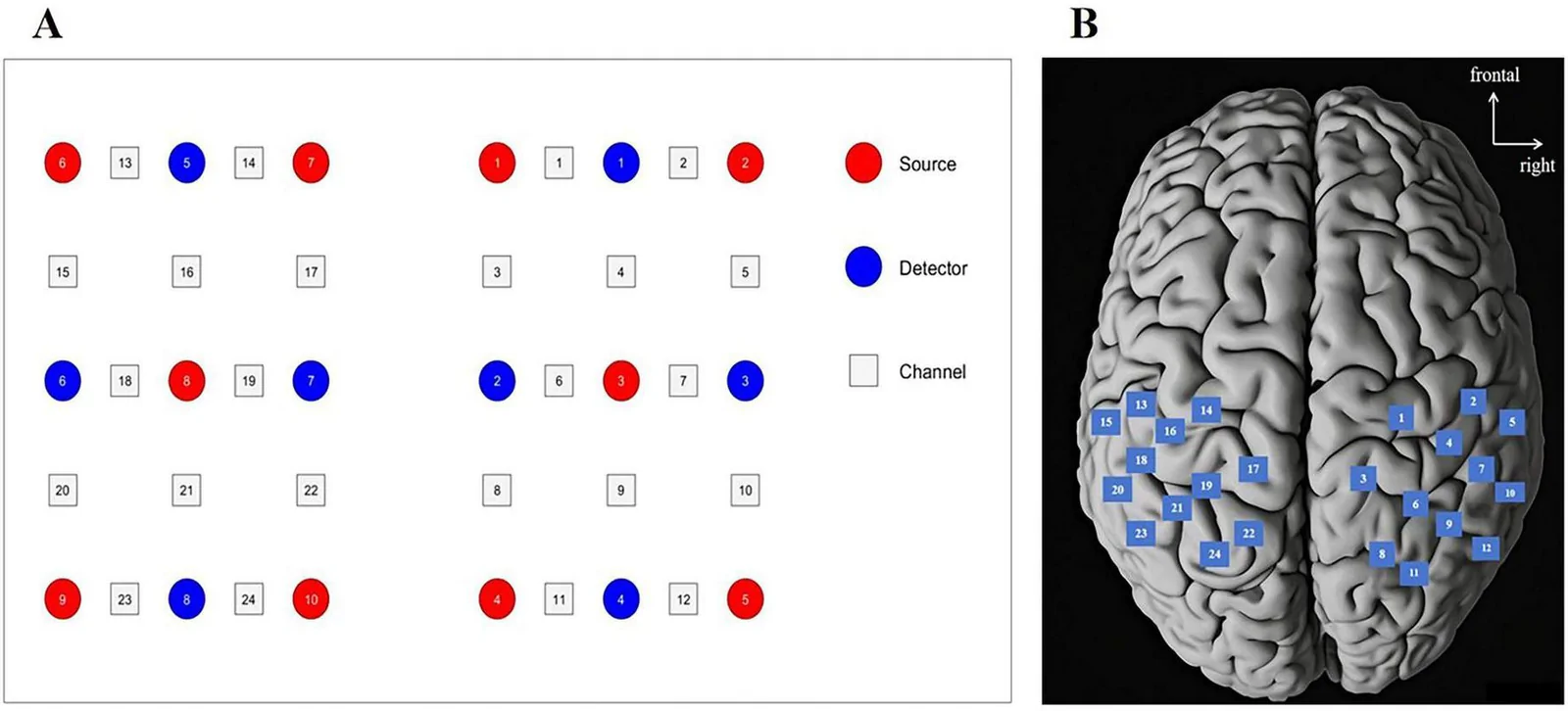
Regions of interest (ROI) channel assignment. **(A)** Locations of optical emitters (red color) and detectors (blue color) within the fNIRS channel. **(B)** Diagram of the fNIRS channel. fNIRS: functional near-infrared spectroscopy.

Cortical oxygenated hemoglobin (HbO) levels, which serve as an indirect indicator of hemodynamic activity in the brain, were monitored throughout all experimental tasks using a wireless, portable fNIRS system with multiple channels (Artinis Medical Systems, The Netherlands). The instrument employs two near-infrared wavelengths (670 nm and 850 nm) and acquires data at a sampling rate of 10 Hz. It is equipped with 10 light sources and 8 detectors, resulting in a total of 24 measurement channels. The specific channel configuration is illustrated in Figure 2.

Specifically, Montreal Neurological Institute (MNI) coordinates and Brodmann areas were determined for each channel based on existing 10/20 system reference information with an interprobe distance of 30 mm. The Cz electrode position for each participant was determined by measuring the intersection of the connection from the nose to the anterior occipital process and the wire with the earlobe of both ears. Furthermore, the Cz electrode’s accuracy was further verified by visual inspection of its alignment along the midsagittal plane to ensure consistent positioning across participants.

The cortical regions of interest (ROIs) included the bilateral primary motor cortex (M1), primary sensory cortex (S1), and somatosensory association cortex (SAC), based on prior evidence of their involvement in KOA-related neural changes (Anders et al., 2004; Bhattacharjee et al., 2021; Sansare et al., 2025). The channel assignments corresponding to each ROI were presented in Table 1.

**TABLE 1:** The fNIRS channel assignments for the ROIs.

ROIs	Left brain	Right brain
S1	13, 16, 17, 18	3, 4, 5
M1	14	1, 2
SAC	19, 21, 22, 24	6, 7, 8, 9, 11

ROIs, regions of interest; S1, primary sensory cortex; M1, primary motor cortex; SAC, somatosensory association cortex.

fNIRS data processing was performed using the Near Infrared Spectroscopy Statistical Parametric Mapping (NIRS-SPM) toolbox in MATLAB (The Mathworks, Natick, MA, USA). Channel quality screening was firstly conducted. Channels with a coefficient of variation greater than 30% of the light intensity signal were determined to be of poor quality and excluded. If more than 50% of the channels within any ROIs were excluded, the participant’s data from that ROIs were considered invalid and not included in the final group analysis. After the remaining qualified data were converted from light intensity to optical density, motion artifacts were processed using the integrated motion correction pipeline within the NIRS-SPM toolbox, which employs a robust algorithm combining moving standard deviation-based detection, spline interpolation, and wavelet denoising. To separate cortical activity from superficial physiological noises such as scalp blood flow, short-channel regression technique was employed. The time series of each long-channel signal was linearly regressed against the signals from its adjacent short-channel, and the regression residuals were used as processed cortical signals for subsequent analysis. A bandpass filter (0.01–0.2 Hz) was subsequently applied to attenuate physiological interference from cardiac, respiratory, and slow drift components (Guan et al., 2024). The modified Beer-Lambert law was used to convert the optical density values into relative changes in HbO concentration.

Following preprocessing, the generalized linear model (GLM) was constructed using the NIRS-SPM toolbox for further analysis. At the individual level, a first-level GLM was constructed for each participant. The design matrix comprised boxcar regressors time-locked to each task trial, with their onset and duration defined by the corresponding event markers. These regressors were convolved with a canonical hemodynamic response function. Resting baseline and recovery periods were included as nuisance regressors, and a high-pass filter was applied to remove low-frequency signal drifts. This model estimated the task-related changes in HbO concentration relative to baseline for each channel. At the group level, for each task, a one-sample *t*-test (*P* < 0.05, FDR corrected) was performed on task-related changes in HbO for all participants within each ROIs to assess whether there was a significant increase in HbO concentration and further statistical analysis.

### 1.6 sEMG data acquisition and preprocessing

Lower limb muscle activity during three walking tasks were recorded using a multi-channel wireless sEMG system (Ultium EMG, Noraxon, USA). Electrodes of sEMG were placed over the most prominent sites of the target muscle bellies in accordance with sEMG for the Non-Invasive Assessment of Muscles guidelines, to capture activity from the vastus medialis (VM), vastus lateralis (VL), and rectus femoris (RF) (Hermens et al., 2000).

sEMG data were processed in batches using a custom MATLAB script. First, the original signal was filtered through a 10–500 Hz bandpass filter (4-order Butterworth) to remove motion artifacts and high-frequency noise. Next, DC offset was removed by deaveraging, and the preprocessed signals were full-wave rectified. The Hilbert transform was used to obtain the signal envelope, and then smoothed by a 6 Hz low-pass filter to obtain a linear envelope. Each muscle signal was normalized to its mean amplitude obtained in the maximum voluntary contraction test. According to the motor task markers, the signal of the stable execution phase in each trial was intercepted (removing the start and stop phases). The root mean square (RMS) and the integrated electromyogram (iEMG) value of each trial were calculated for subsequent statistical analysis.

### 1.7 Clinical scales

Pain intensity in patients with KOA was measured using the VAS, a self-reported tool from 0 to 10 (He et al., 2022). Knee joint function was assessed using the WOMAC (the standardized 24-item Likert version 3.1), which ranges from 0 to 96. It includes a comprehensive assessment of knee pain, stiffness, and function (Gandek, 2015).

### 1.8 Statistical analysis

All statistical procedures were conducted with IBM SPSS Statistics, Version 21.0 (IBM Corp., Armonk, NY, USA). The normality of continuous variables were assessed using the Shapiro-Wilk test. Data following a normal distribution were expressed as mean ± standard deviation; non-normal data were summarized as median and interquartile range. Categorical variables were expressed as counts and percentages. To compare demographic variables between groups, independent samples *t*-tests were applied for normally distributed continuous data, and the Mann-Whitney U test was used for non-normal distributions. Group differences in sex distribution were evaluated using the chi-square test.

For cortical activation analysis, a flexible factorial design was used to create a mixed-effects model with group and task factors. For muscle activation, a mixed ANOVA or nonparametric test (if nonnormal) compared differences between KOA and HC groups across tasks. Pearson correlation coefficients assessed the relationship between cortical activation (HbO concentration) and the score of VAS and WOMAC in patients with KOA, with correlation strengths categorized as low (r < 0.30), moderate (0.30 < r < 0.60), and high (r > 0.60) (Monticone et al., 2018). A significance threshold of 0.05 was applied. The Bonferroni adjustment for multiple comparisons was consistently adopted across all analyses of cerebral ROIs and three walking tasks.

## 2 Results

### 2.1 Demographic data of the participants

The demographic data of the patients with KOA and HCs were presented in Table 2. All enrolled participants completed the entire experiment, and all processed qualified data were included in the analysis. No significant differences were observed between the two groups for any demographic variables (*P* > 0.05).

**TABLE 2:** Demographic data of the patients with knee osteoarthritis and health control groups.

Characteristic	KOA (*n* = 19)	HCs (*n* = 18)	*P*-value
Age (years)	64.16 ± 6.43	62.50 ± 7.03	0.459
Gender (male/female)	14/5	13/5	0.605
Height (m)	1.64 ± 0.06	1.63 ± 0.05	0.788
Weight (kg)	66.76 ± 7.54	64.81 ± 8.83	0.469
BMI (kg/m^2^)	25.95 ± 2.21	24.33 ± 2.57	0.434
VAS	4.47 ± 1.02	/	/
WOMAC	48.42 ± 7.44	/	/

KOA, knee osteoarthritis; HCs, healthy controls; BMI, body mass index; VAS, Visual Analog Scale; WOMAC, Western Ontario and McMaster Universities Osteoarthritis Index.

### 2.2 Differences in cerebral cortical activation between patients with KOA and HC groups

**FIGURE 3:**
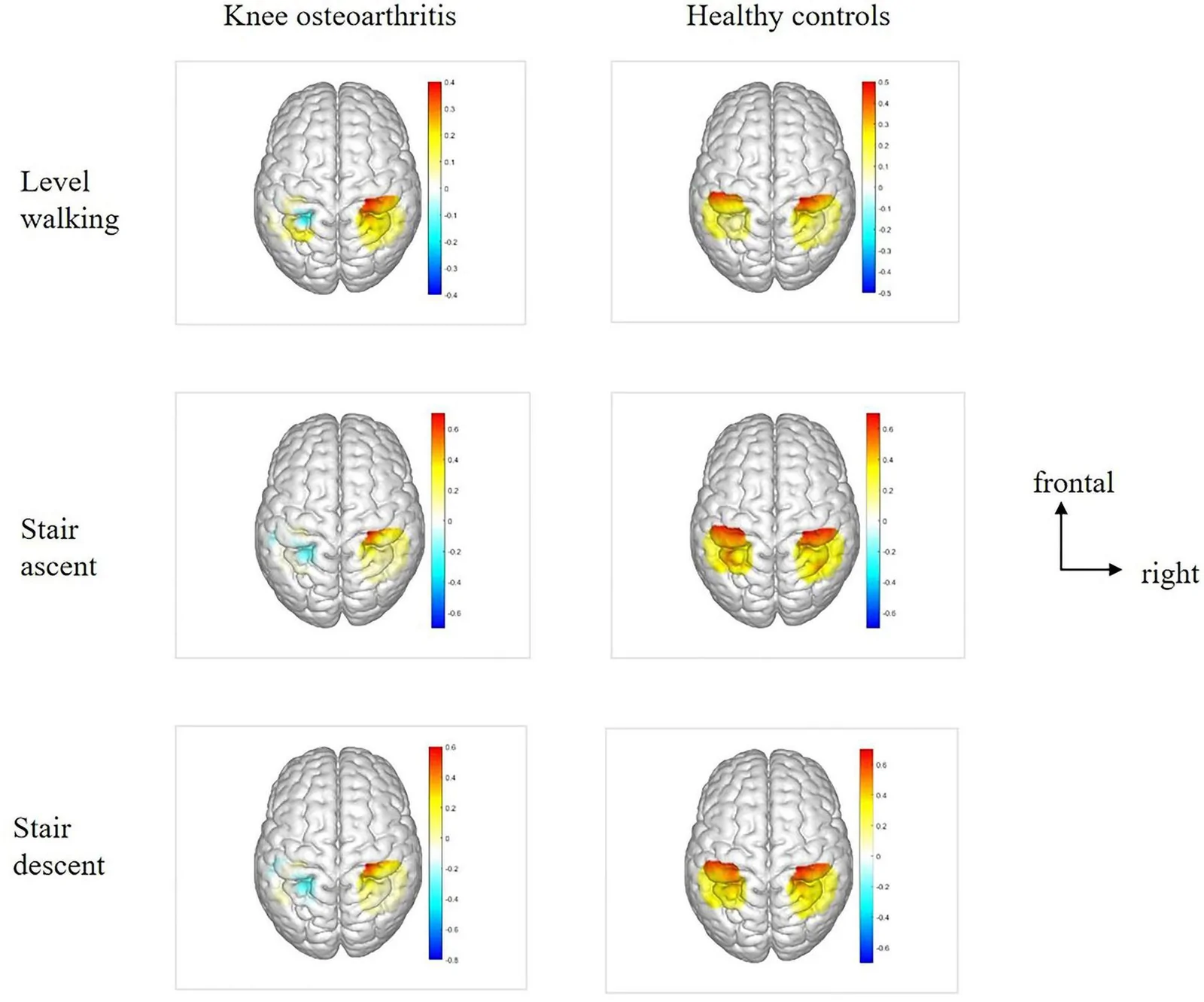
The maps show mean oxyhemoglobin (HbO) levels in patients with knee osteoarthritis and healthy control groups during the three walking tasks. Color transitions from blue to red indicates increasing activation intensity.

Figure 3 displays the cortical HbO concentrations in patients with KOA and HCs across three motor tasks. In the HC group, all walking tasks elicited bilateral activation in M1, S1, and SAC, with no statistically significant interhemispheric differences observed (*P* > 0.05). In KOA participants, during stair ascent, activation in the left M1 (*P* = 0.003, 95% CI [−0.652, −0.153]), S1 (*P* < 0.001, 95% CI [−0.955, −0.355]), and SAC (*P* = 0.009, 95% CI [−0.652, −0.108]) was significantly reduced compared to the right hemisphere (*P* < 0.05); similarly, during stair descent, activation in the left M1 (*P* = 0.007, 95% CI [−0.666, −0.124]), S1 (*P* = 0.011, 95% CI [−1.192, −0.175]), and SAC (*P* = 0.048, 95% CI [−0.735, −0.004]) was significantly reduced compared with the right hemisphere. During level walking, left S1 activation was significantly lower than that on the right side (*P* = 0.002, 95% CI [−0.729, −0.202]), whereas no notable differences were found between hemispheres for M1 (*P* > 0.05, 95% CI [−0.319, 0.131]) and SAC (*P* > 0.05, 95% CI [−0.208, 0.257]).

**FIGURE 4:**
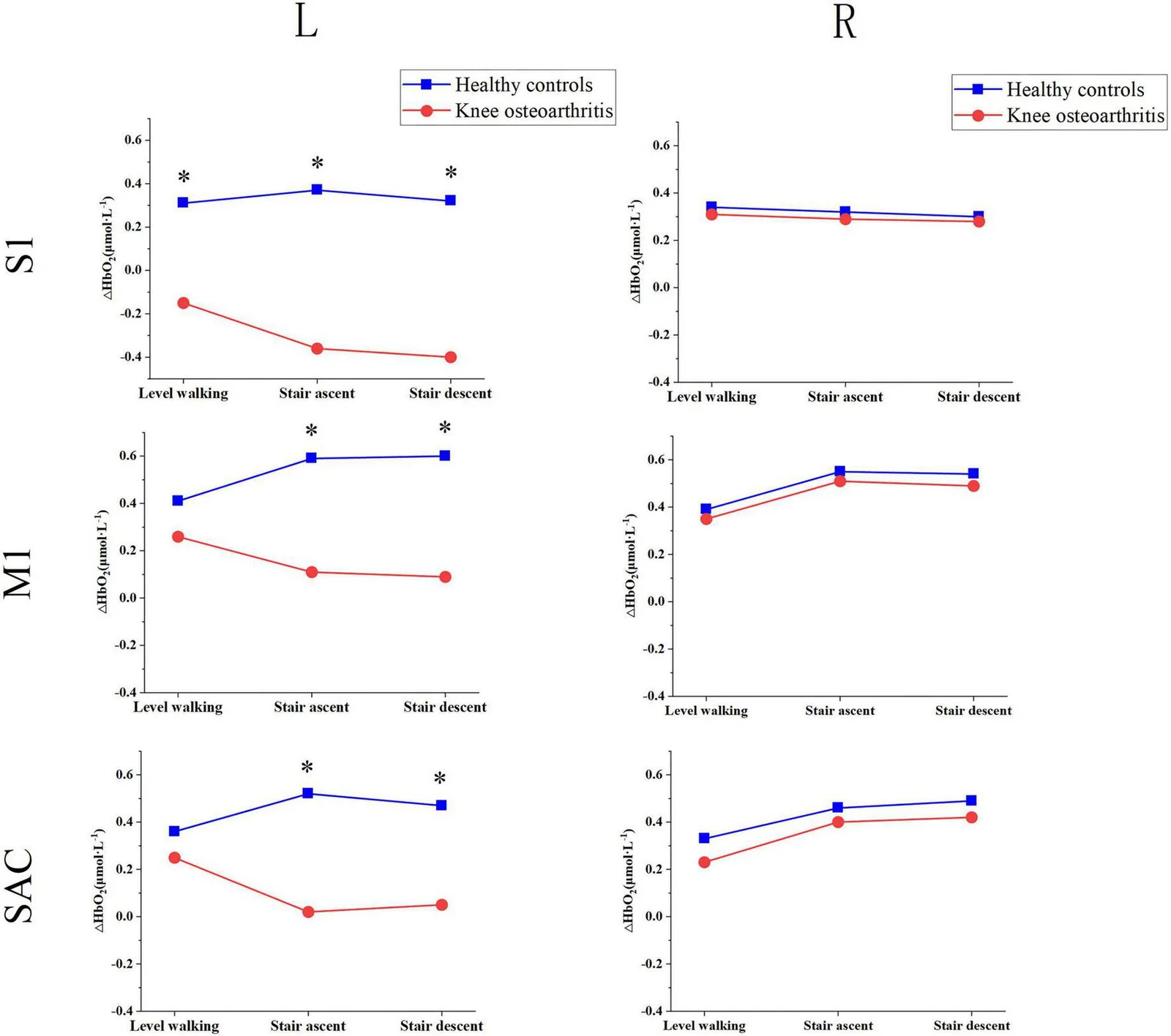
Average cortical activation in patients with knee osteoarthritis and healthy controls. *Compared to health control, *P* < 0.05; S1, primary somatosensory; M1, primary motor; SAC, somatosensory association cortex.

As shown in Figure 4, KOA group showed lower levels of left cortical activation compared with HC group. During stair ascent, significantly lower activation was observed in the left M1 (*P* < 0.001, η^2^ = 0.393, 95% CI [−0.691, −0.277]), S1 (*P* < 0.001, η^2^ = 0.365, 95% CI [−1.069, −0.403]), and SAC (*P* = 0.004, η^2^ = 0.209, 95% CI [−0.836, −0.167]) regions in the KOA group compared to HC group. During stair descent, significantly lower activation was observed in the left M1 (*P* < 0.001, η^2^ = 0.399, 95% CI [−0.691, −0.277]), S1 (*P* = 0.006, η^2^ = 0.198, 95% CI [−1.222, −0.223]), and SAC (*P* = 0.040, η^2^ = 0.115, 95% CI [−0.819, −0.020]) regions in the KOA group compared to HC group. During level walking, left S1 (*P* = 0.002, η^2^ = 0.233, 95% CI [−0.757, −0.176]) activation remained significantly diminished in the KOA cohort, whereas no notable differences were identified between groups for left M1 (*P* > 0.05, η^2^ = 0.070, 95% CI [−0.350, 0.039]) or SAC (*P* > 0.05, η^2^ = 0.008, 95% CI [−0.545, 0.321]). Furthermore, no significant intergroup differences were detected in the right M1, S1, or SAC regions across any of the three walking conditions (*P* > 0.05).

### 2.3 RMS and iEMG values for patients with KOA and HC groups

As shown in Table 3, both the RMS and iEMG values of the lower limb muscles (RF, VL, and VM) in KOA group were smaller than those in the HC group across all three walking tasks, with significant differences (*P* < 0.05) observed particularly during stair ascent and stair descent.

**TABLE 3:** RMS and iEMG values for patients with knee osteoarthritis and health control groups.

Indicator	Muscle	Group	Level walking	Stair ascent	Stair descent
RMS	RF	KOA	15.93 ± 8.53	13.51 ± 7.45*	12.01 ± 6.91*
		HC	19.74 ± 11.20	23.88 ± 11.02	22.76 ± 9.99
	VL	KOA	17.52 ± 9.98	15.32 ± 8.38*	14.41 ± 6.18*
		HC	20.56 ± 9.23	25.45 ± 10.78	26.82 ± 12.12
	VM	KOA	14.40 ± 6.57	11.28 ± 7.30*	12.39 ± 7.72*
		HC	17.63 ± 7.18	21.54 ± 8.16	20.68 ± 9.61
iEMG	RF	KOA	16.25 ± 9.87	13.93 ± 8.14*	12.83 ± 6.25*
		HC	20.17 ± 10.35	22.89 ± 10.86	23.47 ± 14.03
	VL	KOA	16.77 ± 10.46	17.10 ± 9.70*	17.20 ± 10.67*
		HC	21.26 ± 14.64	25.94 ± 14.16	28.11 ± 12.11
	VM	KOA	13.87 ± 8.33	12.58 ± 7.60*	14.11 ± 8.74*
		HC	16.25 ± 9.87	13.93 ± 8.14	12.83 ± 6.25

*Compared with HC, *P* < 0.05; RMS, root mean square; iEMG, integrated electromyogram; KOA, knee osteoarthritis; HCs, healthy controls; RF, rectus femoris; VL, vastus lateralis; VM, vastus medialis.

### 2.4 Correlations between cortical activation and clinical index in patients with KOA

**FIGURE 5:**
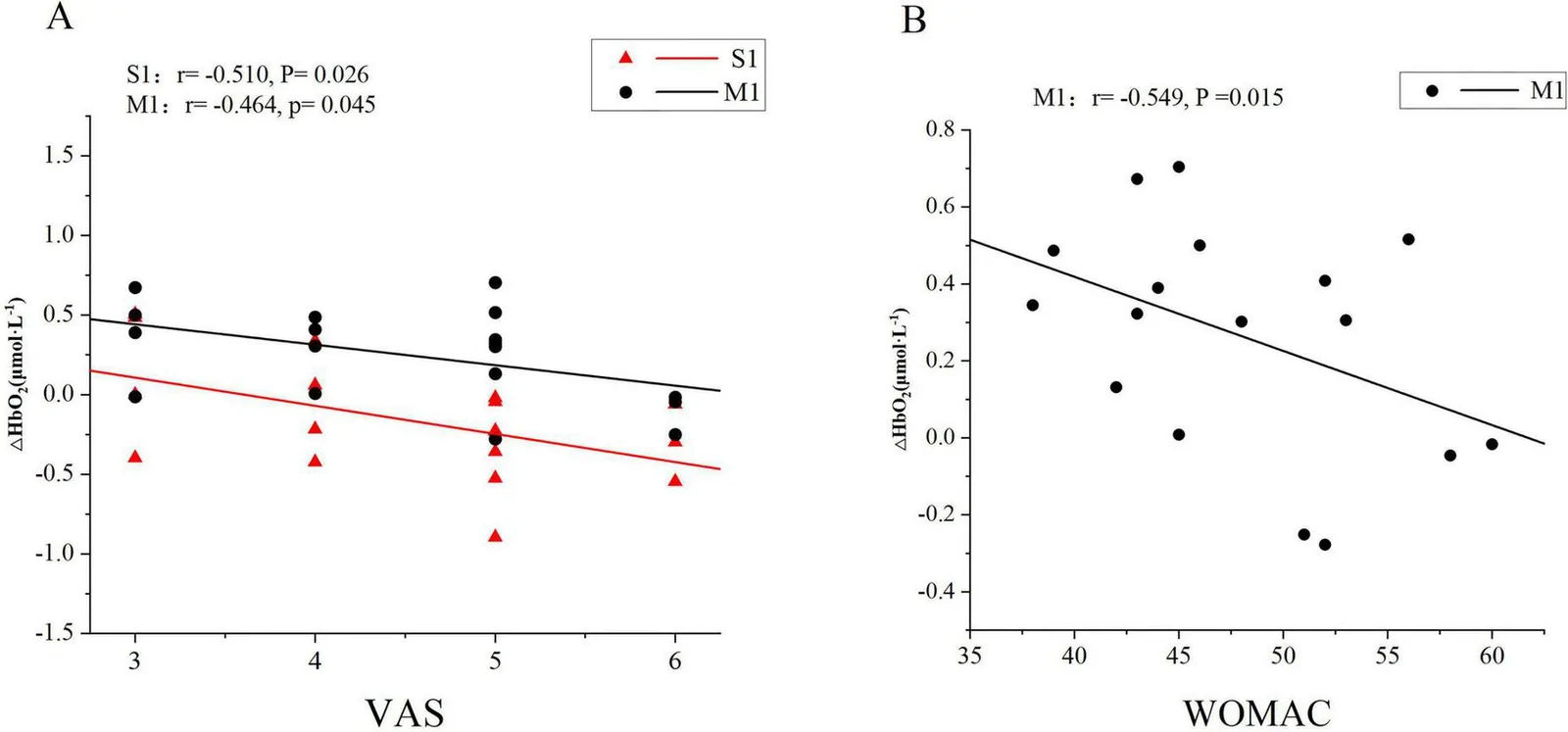
Correlations between cortical activation and **(A)** Visual Analog Scale (VAS) score, or **(B)** Western Ontario and McMaster Universities Osteoarthritis Index (WOMAC) score. S1, primary somatosensory; M1, primary motor.

Correlations between clinical pain indicators and cortical activation revealed that VAS scores were significantly negatively associated with activation in the left M1 (*r* = −0.464, *P* = 0.045) and S1 (*r* = −0.510, *P* = 0.026) during level walking, both indicating moderate correlations (Figure 5A). Regarding functional impairment, the WOMAC score also demonstrated a significant moderate negative correlation with left M1 activation (*r* = −0.549, *P* = 0.015) under level walking condition (Figure 5B). No other significant correlations were observed under other walking conditions. Furthermore, no significant correlations were identified among VAS, WOMAC, and SAC.

## 3 Discussion

We used fNIRS to compare changes in sensorimotor cortical activation between patients with KOA and HCs during three walking tasks. Our findings revealed that, unlike bilateral cortical activation observed in HC group, patients with KOA exhibited reduced activation in the left cortex. Correlation analysis further demonstrated that in patients with KOA, the VAS pain score was significantly negatively correlated with activation in the left M1 and S1, and the WOMAC function score was significantly negatively correlated with the left M1.

Specifically, in the HC group, bilateral activation of M1, S1, and SAC was observed across different walking tasks, with no significant difference in activation intensity between the two hemispheres. This bilateral symmetric activation in the M1, S1, and SAC regions observed in HC is consistent with previous study (Bishnoi et al., 2024). This reflects that during normal, highly automated walking, the CNS coordinates and integrates symmetric and rhythmic motor commands and sensory feedback for both lower limbs through the collaborative activity of bilateral hemispheric networks. This phenomenon has been consistently confirmed in many kinds of motion studies. Jang et al. (2017) using fNIRS during bilateral arm raising tasks, reported significant bilateral activation across sensorimotor and premotor regions without notable lateralization. Similarly, Jordon et al. (2022) demonstrated through fMRI that bilateral bridge tasks robustly engage motor areas bilaterally in healthy participants. Although unilateral bridging elicited predominantly contralateral activation, the authors highlighted that even unilateral lumbopelvic motions likely involve cooperative bilateral neural control mechanisms due to the requirement for multi-segmental coordination and core stabilization (Jordon et al., 2022). However, existing research confirms that the dominant limb has a unique pattern of interhemispheric inhibition that is not found on the non-dominant limb (Vidal et al., 2014). Therefore, in order to eliminate leg dominance as a confounding factor, only right-leg-dominant participants were included in both the KOA and HC groups in our study.

In contrast, patients with KOA showed significant brain activation asymmetry and generally lower cortical activation compared with the HC group. During level walking, although activation in the M1 and SAC regions remained bilaterally symmetric, the activation level of the left S1 responsible for processing somatosensory information was significantly lower than that in the right S1. Furthermore, during level walking, activation in the left S1 was significantly lower in the KOA group than in the HC group, while no significant differences were observed in M1 and SAC activation in two groups. The S1 region, located in the postcentral gyrus and is a key node for receiving and processing sensory signals from the periphery (Tatiana et al., 2004; Sansare et al., 2025). We speculate that this may result from dysfunctional sensory processing due to chronic pain, reduced joint proprioceptive input, and/or abnormal afferent signaling caused by KOA. This may have induced long-term inhibitory plastic changes in the contralateral S1 region, which were not observed in M1 and SAC. This abnormality in sensory processing is likely a precursor to motor control impairments (Sansare et al., 2025).

Importantly, during stair climbing tasks that impose greater motor load and coordination demands, patients with KOA group exhibited more extensive and pronounced imbalances in brain activation. Activation in the left S1 significantly lower than that in the right, but activation in the left M1 and SAC was also significantly reduced. Moreover, during stair negotiation, the activation levels of the left M1, S1, and SAC in the KOA group were significantly smaller than those in the HC group. These results strongly suggest that as task difficulty increases, placing higher demands on motor control, balance, and sensory integration, compensatory mechanisms within the central nervous system of patients with KOA may be challenged or dysfunctional. Compared to level walking, stair negotiation features larger knee flexion and extension angles, higher joint contact pressure and heavier load on quadriceps. Increased joint stress aggravates cartilage wear and easily triggers or aggravates pain and mobility limitation in KOA patients, which may further trigger more obvious inhibition of sensorimotor cortical activation.

The observed reduction in M1 activation may be related to the mechanism of “pain-related neural inhibition,” whereby chronic knee pain selectively inhibits the activity of M1 neurons controlling the affected limb via inhibitory circuits at both spinal and cortical levels, thereby reducing motor output to avoid pain (Antonella et al., 2025; Lee et al., 2025). Concurrently, insufficient activation of the SAC suggests impairment in processes of sensory integration and motor planning. This central activation deficit aligns with the reduced muscle activation recorded via sEMG, particularly during stair climbing, suggesting a potential association between “weakened central command” and “impaired peripheral execution.”

Furthermore, our findings revealed that, during level walking, higher VAS scores in patients with right-sided KOA were associated with lower activation levels in the left M1 and S1. Similarly, higher WOMAC scores were associated with reduced activation levels in the left M1. The reduction in M1 activity can be viewed as a central adaptive protective mechanism, reducing motor output to avoid exacerbating pain, but ultimately may be related to impaired motor function. Meanwhile, diminished activation in S1 reflects aberrant sensory processing under chronic pain conditions and may directly relate to the proprioceptive deficits commonly observed in patients with KOA (Abdullah et al., 2023). These findings provide compelling empirical evidence elucidating the intrinsic relationship between chronic pain and functional alterations in the CNS in KOA.

Interestingly, these correlation results exhibited notable task and brain region specificity. Significant correlations were only observed during level walking but not during stair ascent and stair descent. A plausible explanation is that the clinical metrics such as VAS and WOMAC reflect average symptom levels over time. Level walking, being frequent and behaviorally consistent, yields stable cortical activation patterns that correlate with long-term pain and functional limitations. In contrast, during more challenging stair negotiation tasks, factors such as fear avoidance, attentional diversion, and greater biomechanical variability may obscure the direct correlation between pain and cortical activation (Anoohya et al., 2019; Gabriel Peixoto Leão et al., 2021). These correlative findings suggest that contralateral cortex (especially M1), as measured by fNIRS, may not only serve as an objective neurophysiological indicator of disease severity but also a potentially biomarker for predicting functional performance. However, these findings are preliminary and require validation in larger cohorts. Nonetheless, this provides a direct theoretical basis for developing novel neuromodulatory interventions (e.g., transcranial direct current stimulation targeting contralateral M1).

This study has several limitations. This is a cross-sectional study that cannot establish causal relationships between cortical activity and clinical symptoms. Furthermore, the lack of stratified analyses based on KL grade and gender may have masked subgroup differences. Finally, the relatively small number of participants may limit statistical power for detecting smaller effect sizes and reduce the generalizability of our findings to the broader KOA population. Future studies should adopt longitudinal designs to assess synchronous changes in clinical symptoms and cortical activity before and after intervention, ideally with larger, stratified samples combined with biomechanical analysis and neuromodulation techniques. Such studies will more accurately elucidate the core mechanisms of KOA and identify precise intervention targets.

## 4 Conclusion

In summary, patients with KOA demonstrated reduced activation in sensory and motor cortices during level walking, and stair climbing tasks, with these deficits becoming more pronounced as the task demands increased. Furthermore, pain severity and functional limitations were negatively correlated with activation levels in the contralateral primary motor and primary sensory cortices. These findings highlight altered central processing as a key component of KOA pathophysiology and provide novel neurophysiological evidence supporting the role of cortical mechanisms in chronic pain and motor dysfunction. Future large-scale, longitudinal studies are necessary to confirm these results and to establish their clinical utility in guiding centrally oriented rehabilitation interventions.

## Data Availability

The raw data supporting the conclusions of this article will be made available by the authors, without undue reservation.
